# YUCCA4 overexpression modulates auxin biosynthesis and transport and influences plant growth and development via crosstalk with abscisic acid in Arabidopsis thaliana

**DOI:** 10.1590/1678-4685-GMB-2019-0221

**Published:** 2020-02-17

**Authors:** Aarón Giovanni Munguía-Rodríguez, Jesús Salvador López-Bucio, León Francisco Ruiz-Herrera, Randy Ortiz-Castro, Ángel Arturo Guevara-García, Nayelli Marsch-Martínez, Yazmín Carreón-Abud, José López-Bucio, Miguel Martínez-Trujillo

**Affiliations:** ^1^ Universidad Michoacana de San Nicolás de Hidalgo Universidad Michoacana de San Nicolás de Hidalgo Instituto de Investigaciones Químico-Biológicas Michoacán Mexico Instituto de Investigaciones Químico-Biológicas, Universidad Michoacana de San Nicolás de Hidalgo, Edificio B3, Ciudad Universitaria. Morelia, Michoacán, Mexico.; ^2^ Universidad Michoacana de San Nicolás de Hidalgo Universidad Michoacana de San Nicolás de Hidalgo CONACYT-Instituto de Investigaciones Químico-Biológicas Changzhou Mexico CONACYT-Instituto de Investigaciones Químico-Biológicas, Universidad Michoacana de San Nicolás de Hidalgo, Edificio B3, Ciudad Universitaria. Morelia, Michoacán, Mexico.; ^3^ CONACYT-Red de Estudios Moleculares Avanzados CONACYT-Red de Estudios Moleculares Avanzados Instituto de Ecología A. C. Carretera antigua a Coatepec 351 Veracruz Mexico CONACYT-Red de Estudios Moleculares Avanzados, Instituto de Ecología A. C. Carretera antigua a Coatepec 351, Colonia El Haya. Xalapa, Veracruz, Mexico.; ^4^ Universidad Nacional Autónoma de México Universidad Nacional Autónoma de México Instituto de Biotecnología Morelos Mexico Instituto de Biotecnología, Universidad Nacional Autónoma de México, Cuernavaca, Morelos, Mexico.; ^5^ Departamento de Biotecnología y Bioquímica Departamento de Biotecnología y Bioquímica Centro de Investigación y Estudios Avanzados Guanajuato Mexico Departamento de Biotecnología y Bioquímica, Centro de Investigación y Estudios Avanzados, Unidad Irapuato, Irapuato, Guanajuato, Mexico.; ^6^ Universidad Michoacana de San Nicolás de Hidalgo Universidad Michoacana de San Nicolás de Hidalgo Facultad de Biología Michoacán Mexico Facultad de Biología, Universidad Michoacana de San Nicolás de Hidalgo. Morelia, Michoacán, Mexico.

**Keywords:** Arabidopsis, auxin, abscisic acid, YUCCA4, root growth, germination

## Abstract

Auxin regulates a plethora of events during plant growth and development, acting in concert with other phytohormones. *YUCCA* genes encode flavin monooxygenases that function in tryptophan-dependent auxin biosynthesis. To understand the contribution of the *YUCCA4* (*YUC4*) gene on auxin homeostasis, plant growth and interaction with abscisic acid (ABA) signaling, *35S::YUC4* seedlings were generated, which showed elongated hypocotyls with hyponastic leaves and changes in root system architecture that correlate with enhanced auxin responsive gene expression. Differential expression of PIN1, 2, 3 and 7 auxin transporters was detected in roots of *YUC4* overexpressing seedlings compared to the wild-type: PIN1 was down-regulated whereas PIN2, PIN3 and PIN7 were up-regulated. Noteworthy, *35S::YUC4* lines showed enhanced sensitivity to ABA on seed germination and post-embryonic root growth, involving ABI4 transcription factor. The auxin reporter genes *DR5::GUS, DR5::GFP* and *BA3::GUS* further revealed that abscisic acid impairs auxin responses in *35S::YUC4* seedlings. Our results indicate that *YUC4* overexpression influences several aspects of auxin homeostasis and reveal the critical roles of ABI4 during auxin-ABA interaction in germination and primary root growth.

## Introduction

The phytohormone auxin (indole-3-acetic acid, IAA) plays a role in many aspects of plant growth and development, including cell division, growth and differentiation. It also mediates adaptation to biotic and abiotic stress (Ghanashyam and Jain, 2009; Rahman, 2013). These functions require coordinated IAA biosynthesis, degradation, conjugation, transport and signaling for which specific genes and proteins have been identified in *Arabidopsis* and crops. Auxin biosynthesis mainly occurs in developing tissues such as cotyledons, expanding leaves and root tips (Ljung *et al.*, 2001), and arises via tryptophan (Trp)-independent and Trp-dependent pathways (Zhao, 2010). In the second case, Trp is first converted into indole-3-pyruvic acid by TRYPTOPHAN AMINOTRANSFERASE OF ARABIDOPSIS1/TRYPTOPHAN AMINOTRANSFERASE RELATED (TAA1/TAR) enzymes (Kasahara, 2015). Subsequently, enzymes of the YUCCA family of flavin-containing mono-oxygenases (FMOs) catalyze the conversion of indole-pyruvic acid (IPA) into IAA. This two-step auxin biosynthesis pathway is highly conserved throughout the plant kingdom and is essential for almost all of the major developmental transitions and whole plant functioning (Zhao, 2012).

The *YUC* gene family has been identified in several plant species, it includes eleven members in *Arabidopsis* (Cheng *et al.*, 2006), seven in rice (Yamamoto *et al.*, 2007), six in tomato (Exposito-Rodriguez *et al.*, 2011), eight in strawberry (Liu *et al.*, 2014), twelve in poplar (Ye *et al.*, 2009) and ten in cucumber (Yan *et al.*, 2016). Disruption of a single *YUC* gene in *Arabidopsis* shows no obvious phenotypical alterations, which implicates functional redundancy. However, double, triple and quadruple mutants show abnormalities in different developmental and tissue specific contexts (Cheng *et al.*, 2006). On the other hand, gain-of-function YUC plants exhibit phenotypes consistent with auxin overproduction. From the 11 *YUC* homologues in *Arabidopsis*, overexpression of YUCAA1,3,5,6,7,8, 9 has been studied under different contexts (Zhao *et al.*, 2001; Woodward *et al.*, 2005; Kim *et al.*, 2007; Lee *et al.*, 2012; Hentrich *et al.*, 2013b; Chen *et al.*, 2014a; Cha *et al.*, 2015). However, overexpression of YUCCA4 gene and its impact on plant development has not been studied to date; a previous work only reports a line called *thread,* generated by activation tag inserts in *Arabidopsis* using the maize (*Zea mays*) En-I transposon system (Marsch-Martinez *et al.*, 2002).[Bibr B34]

Auxin is distributed via two spatially separated transport pathways: in the phloem it moves by mass flow (Muday and DeLong, 2001; Michniewicz *et al.*, 2007), while in other tissues, it is transported cell-to-cell through the PIN-FORMED (PIN) proteins, distributed differentially within cell membranes of transporting tissues (Galweiler *et al.*, 1998; Muller *et al.*, 1998; Krecek *et al.*, 2009). These transport systems ensure auxin redistribution according to the cell physiological and developmental status, and at the same time enable rapid growth and patterning responses.

Auxin is perceived by TIR1 and related AFB1, AFB2 and AFB3 protein receptors, associated with the SCF complex (Benjamins and Scheres, 2008). Auxin-responsive genes are commonly activated by specific transcription factors termed auxin-response factors (ARFs) through binding to auxin response elements (AREs) present in their promoters (Chandler, 2016). By contrast, the AUX/IAA repressors, negatively regulate auxin responses via interaction with ARFs (Hagen and Guilfoyle, 2002). Auxin acts as a glue to attach the AUX/IAA proteins with SCFTIR1, resulting in ubiquitination and degradation of the AUX/IAA repressors via the proteasome (Quint and Gray, 2006).

In addition to its importance towards understanding hormonal-dependent regulation of plant growth and development, how auxin interacts with abscisic acid (ABA) is a question of growing interest owing its role in adaptation to environmental stress (Kim *et al.*, 2013; Ke *et al.*, 2015; Tiwari *et al.*, 2017). ABA regulates embryo and seed development, seed dormancy, germination, senescence, vegetative growth, lateral root development, and drought tolerance (Finkelstein *et al.*, 2002; De Smet *et al.*, 2003). ABA synthesis takes place in vasculature, stomata and in seeds, where it promotes dormancy and blocks germination (Boursiac *et al.*, 2013). The cells perceive ABA through various receptor families, some of them localized into the nucleus. Currently, the best established ABA signaling model involves the soluble PYR/PYL/RCAR receptors, and downstream acting PP2C phosphatases that directly regulate SnRK2 kinases, controlling the transcription factors that finally regulate expression of ABA responsive genes (Cutler *et al.*, 2010).

Here, we generated and characterized *Arabidopsis thaliana* lines that overexpress the *YUC4* gene under transcriptional control of the CaMV 35S promoter (*35S::YUC4*). An analysis of these lines enabled not only to establish the functionality of the corresponding coding sequence, but also to perform a detailed investigation on growth and development related to auxin biosynthesis and transport, and characterization of the auxin-ABA crosstalk that influences germination and early plant growth.

## Materials and Methods

### Generation of *YUCCA4* overexpressing lines

The *YUC4* coding sequence was amplified by PCR and then cloned into the vector pENTR/D-TOPO® according to the manufacturer’s protocol (Thermo-Fisher). Primers for cDNA amplification were forward 5-CAC CAT GGG CAC TTG TAG AGA A-3 and reverse 5-TCA CAT ATA CAT ATA CAC ATT GAC-3. PCR product clones were confirmed by nucleotide sequencing and mobilized by recombination into the binary vector pEarleyGate100. The resulting vector was transferred to the *Agrobacterium tumefaciens* strain pGV2260 to perform *Agrobacterium*-mediated transformation of *Arabidopsis* (ecotype Col-0) plants using the modified floral dip method (Martinez-Trujillo *et al.*, 2004). T1 seedlings were selected on MS medium containing 50 μg/mL of glufosinate ammonium (BASTA). BASTA-resistant T1 seedlings were transferred to soil and allowed to self-pollinate to generate T2 plants. The resistant T2 seedlings with 3:1 segregation of resistance were transferred to soil to obtain homozygous T3 seedlings from individual lines.

### Plant material and growth conditions

*Arabidopsis thaliana* lines used were Col-0 (WT), the transgenic *Arabidopsis* lines *DR5::GUS* (Ulmasov *et al.*, 1997), *BA3::GUS* (Oono *et al.*, 1998), *HS::AXR3NT-GUS* (Gray *et al.*, 2001), *ABI4::GUS* (Shkolnik-Inbar and Bar-Zvi, 2010), *PIN1::PIN1-GFP* (Benková *et al.*, 2003), *PIN2::PIN2-GFP*, *PIN3::PIN3-GFP*, *PIN7::PIN7-GFP* (Blilou *et al.*, 2005) and the mutant line *abi4* (Finkelstein, 1994), Crosses were made between reporter lines and *35S::YUC4*; F3 populations from the crosses were screened for auxin overproducing phenotypes in shoots of plants harboring the marker constructs; homozygous lines were used in subsequent experiments. Seeds were surface sterilized with 95% ethanol (v/v) for 5 min and 20% bleach (v/v) for 7 min and washed five times in 1 ml of sterile distilled water. Seeds were vernalized for 2 days at 4 °C and placed into plates containing 0.2x solidified MS medium prepared with MS basal salts (Murashige and Skoog Basal Salts Mixture, Sigma Aldrich), 1% agar (Phytagar Gibco-BRL), and 1% sucrose (Sigma-Aldrich). Plates were vertically placed at an angle of 65° to allow root growth along the agar surface and to allow aerial growth of the hypocotyls, into a plant growth chamber (Percival AR-95L) with a photoperiod of 16 h of light/8 h of darkness, light intensity of 105 μmol m^-2^ s^-1^ and temperature of 22 °C.

### Chemicals

NPA and ABA were purchased from Sigma and dissolved in dimethyl sulfoxide (DMSO). In control treatments, DMSO was used in equal amounts as present in the greatest concentration of each compound tested.

### Analysis of growth

*Arabidopsis* roots and hypocotyls were analyzed using a stereoscopic microscope (Leica MZ6). Images were captured with a Samsung SCC 131-A digital color camera adapted to the microscope. Primary root length was determined for each root using a ruler. Lateral root number was determined by counting the lateral roots per seedling, and lateral root density was calculated by dividing the lateral root number by the primary root length for each analyzed seedling. Hypocotyl length was determined from images using the software NIH ImageJ version 1.48 (Schneider *et al.*, 2012). For all experiments with WT and transgenic lines, the overall data were statistically analyzed using the SPSS 10 program.

### Free IAA determination

Whole seedlings were grown on agar solidified 0.2x MS medium for 10 d, then collected and frozen in liquid N_2_. 100 mg of tissue was pooled per sample. IAA was quantified using the Varian Saturn 2000 GC-MS/MS system as previously described (Pollmann *et al.*, 2009).

### Histochemical analysis

For histochemical analysis of β-glucuronidase, *Arabidopsis* seedlings were incubated overnight at 37 °C in a GUS reaction buffer (0.5 mg mL^-1^ 5-bromo-4-chloro-3-indolyl-β-D-glucuronide in 100 mM sodium phosphate, pH7). The stained plants were cleared with 0.24 N in 20% HCl (v/v) methanol and incubated for 60 min at 62 °C. The solution was substituted by 7% NaOH (w/v) in 60% ethanol (v/v) for 20 min at room temperature. Plants were hydrated with ethanol treatments at 40, 20 and 10% (v/v) for 24 h each, and fixed in 50% glycerol (v/v). The processed roots were placed on glass slides and sealed with commercial nail varnish. For each marker line and for each treatment, at least 15 transgenic plants were analyzed.

### Seed germination assays

For germination assay, seeds from WT, *35S::YUCCA4*, *abi4* and *abi4/35S::YUCCA4* were disinfected and placed into 0.2x MS medium supplemented with DMSO, 0.5, 1 and 2 μM ABA, and incubated in a plant growth chamber to register germination at the time when radicle was completely emerged.

### Northern blotting

For RNA hybridization analysis, 10 d seedlings were grinded in liquid N_2_, total RNA was extracted from 50 mg of grinded tissue using TRIzol according to the manufacturers protocol (Invitrogen). RNA (10 μg) was separated in 1.2% formaldehyde agarose gel electrophoresis according to the protocol adapted from Rneasy Mini Handbook (QIAGEN), transferred to Hybond-N nylon membrane (GE Healthcare) and fixed in an UV crosslinker at 70,000 microjoules/cm^2^. Probes were ^32^P radiolabeled with α-^32^P dCTP (Perkin Elmer Life Science Inc.) using Klenow DNA polymerase I according to the protocol of the manufacturer (New England Biolabs). Membranes containing RNA were hybridized for 4 h with the probes tested and washed with a sodium chloride solution (7.5 mM)/sodium citrate (8.75 mM). The probe was detected after 8 h of exposure in an X-Ray film (GE Healthcare). The assayed probes were amplified by PCR reactions from DNA using the indicated oligonucleotides, YUC4 forward 5 GGAAATTCCGGTATGGAGGT 3’ and reverse 5’ GCTCAATTGGTCCGGTCTTA 3’.

### Data materials availability

Plant lines reported are available for research purposes.

## Results

### *35S::YUC4 Arabidopsis* plants show phenotypes related to auxin overproduction

**Figure 1:**
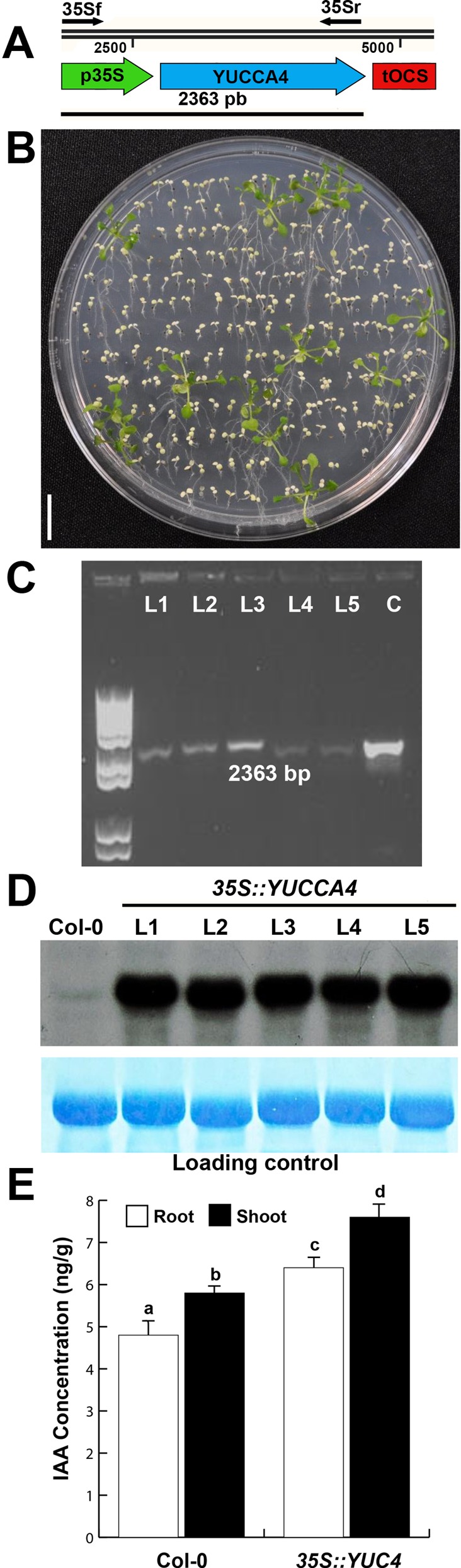
Generation of *35S::YUC4* transgenic plants. (A) Fragment of plasmid map carrying *YUC4* sequence under CaMV35S promoter. Location of forward primer on 35S and reverse on *YUC4* are shown as well as length of flanking sequence. (B) Plants grown on MS media supplemented with glufosinate-ammonium and showing resistance. Bar = 1 cm. (C) PCR gel of five transformed plants (L1-5) showing bands of 2363 bp corresponding to *YUC4* gene and 35S promoter. Line C shows a band from a PCR using cloned plasmid as control. (D) Northern blot indicating transcription levels of *YUC4* in Col-0 and five *35S::YUC4* (L1-5) lines. (E) IAA levels in roots and shoots of Col-0 and *35S::YUC4* L1 in 10 dag plants determined by GC-MS. Bars in (E) show standard errors and different letters indicate statistical differences at *P < 0.05*.

The cDNA of *YUC4* gene was cloned under control of the constitutive CaMV 35S promoter (Figure 1A). Seventeen transformed plants from independent transformation events were selected from glufosinate ammonium (Figure 1B) and five of them were molecularly characterized (Figure 1C). To corroborate the *YUC4* overexpression in all five lines RNA hybridization via Northern blotting was performed; all selected lines showed higher levels of *YUC4* expression than the WT (Figure 1D). Quantification of free IAA content in seedlings of WT and the now denominated *35S::YUC4* line indicated a roughly 25% increase of IAA level in both roots and shoots (Figure 1E). The determined IAA proportion was conserved in all the lines used in this work (Figure S1).

Noteworthy, the *35S::YUC4* transgenic plants exhibited auxin-related phenotypes including epinastic cotyledons and elongated hypocotyls. Adult plants showed characteristic twisted cauline leaves, narrow rosette leaves with long petioles and increased apical dominance and this phenotype was common to the initially identified seventeen lines (Data not shown). *35S::YUC4* seedlings also developed longer and narrower primary roots, and produced more lateral roots that the WT. Due to this combined situation, the lateral root density of the WT and *YUC4* overexpressing seedlings was comparable (Figure S2). Thus, overexpression of *YUC4* promotes hypocotyl and root elongation and lead plants to develop more exploratory root systems, all consistent with changes in auxin homeostasis.

### Overexpression of *YUCCA4* enhances auxin responsiveness and modulates auxin transporters

**Figure 2:**
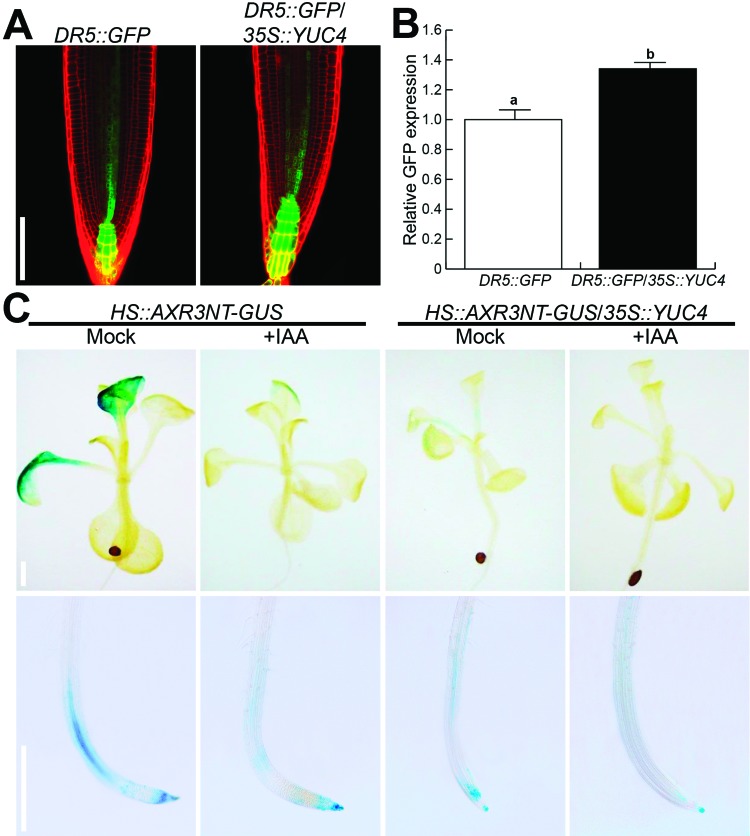
The *Arabidopsis thaliana 35S::YUC4* seedlings show an increased auxin response. (A) *DR5::GFP* in root tips of WT and *DR5::GFP*/*35S::YUC4* seedlings, (B) Relative quantification of GFP fluorescence (n = 10 ? standard error), different letters indicate statistical differences at *P < 0.05*. (C) *HS::AXR3NT-GUS* expression in shoots and roots of WT and *YUC4* overexpressing seedlings. Seedlings were germinated and grown 10d on 0.2X MS medium, transferred to 0.2X MS liquid medium and heat shocked for 2 h at 37 °C to induce expression of the transgene. Seedlings then were transferred to 20 °C medium containing mock and 2 μM IAA and incubated for 1 hr before staining for β-glucuronidase activity. Photographs show representative individuals from at least 10 stained seedlings. Scale bar in A 100 μm; scale bars in C 500 μm. The experiment was repeated three times with similar results.

To investigate if the observed changes in *35S::YUC4* seedlings could be related to an altered auxin response and/or transport, different genetic markers were mobilized into *35S::YUC4,* via outcrossing. The auxin reporter gene *DR5::GFP* showed a higher expression in primary root tips of *35S::YUC4* seedlings than in the WT (Figure 2A, 2B). Next, we evaluated the effect of overexpression of *YUC4* on Aux/IAA degradation. WT and *35S::YUC4* seedlings expressing the *HS::AXR3NT-GUS* (Gray *et al.*, 2001) gene construct were heat shocked at 37 °C for 2 h. After heat shock, seedlings were incubated with and without IAA for a subsequent GUS histochemical detection. In WT seedlings, blue coloration was observed showing AXR3 localization in petioles, root vasculature and root meristem; such coloration was decreased in control treatment with IAA; a similar behavior was observed in the case of *HS::AXR3NT-GUS/35S::YUC4* and the expression was further decreased with IAA (Figure 2C), those results suggest an increased degradation of AXR3 in *35S::YUC4*.

**Figure 3:**
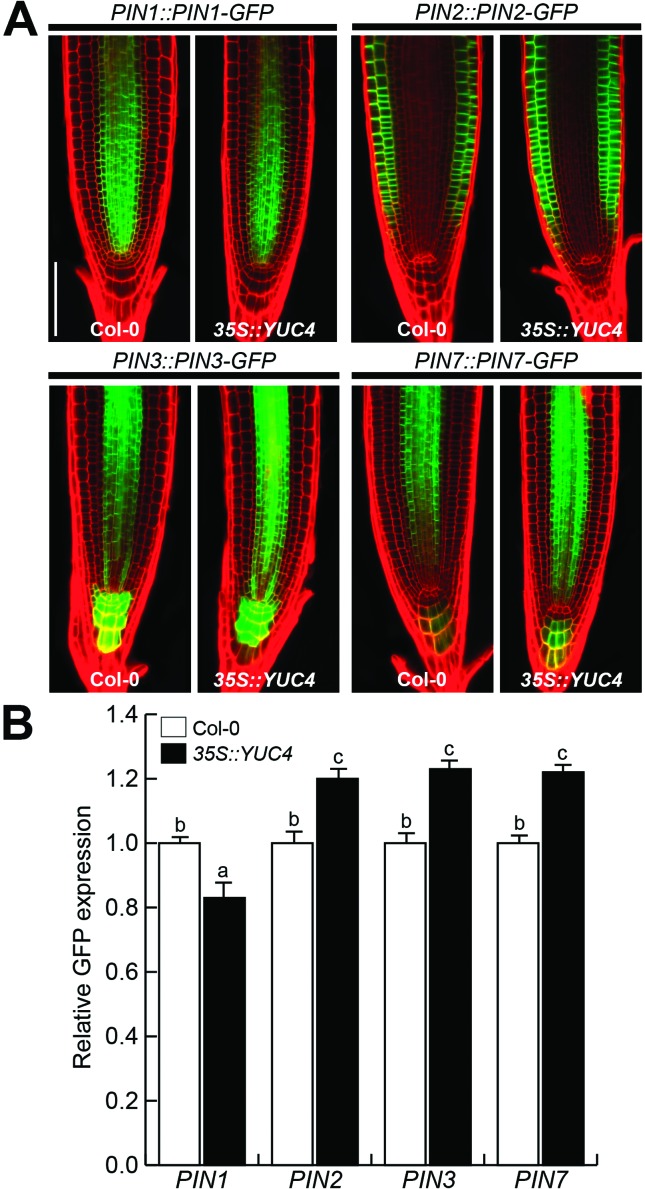
Expression of PIN auxin transporters in WT and *35S::YUC4* seedlings. (A) Confocal microscopy images of WT and *35S::YUC4* seedlings showing *PIN1::PIN1-GFP*, *PIN2::PIN2-GFP*, *PIN3::PIN3-GFP* and *PIN7::PIN-GFP* fluorescence. Bar = 100 μM. (B) Quantification of relative GFP expression of PIN transporters in WT and *35S::YUC4* backgrounds. Plants were grown on MS 0.2X and analyzed at 10 d. Bars in graphics indicate standard error and different letters indicate statistical differences at *P = 0.05*. The analysis was repeated three times with similar results.

PIN auxin transporters mediate IAA distribution within root tissues (Adamowski and Friml, 2015). To evaluate whether PIN auxin transporters are influenced by auxin overproduction, we crossed *35S::YUC4* plants with pollen of plants carrying PIN-GFP protein fusions (Benková *et al.*, 2003; Vieten *et al.*, 2005), and the expression was analyzed in roots. *PIN1* is expressed at the basal side of stele and endodermis in the WT, and a reduction of its expression is observed in *35S::YUC4* seedlings (Figure 3A). *PIN2* expression is localized in membranes of cortical and epidermal cells in WT plants and it was induced in *35S::YUC4* seedlings; similarly both *PIN3* and *PIN7* that are expressed in columella and stele of the elongation zone of the primary root showed an enhanced expression in *35S::YUC4* transgenic line (Figure 3A, 3B). From these results, we conclude that overexpression of *YUC4* and its consequent auxin overproduction differentially modulate expression of PIN proteins in the *Arabidopsis* primary root.

### Inhibition of auxin transport normalizes hypocotyl elongation and auxin accumulation in *35S::YUC4* seedlings

**Figure 4:**
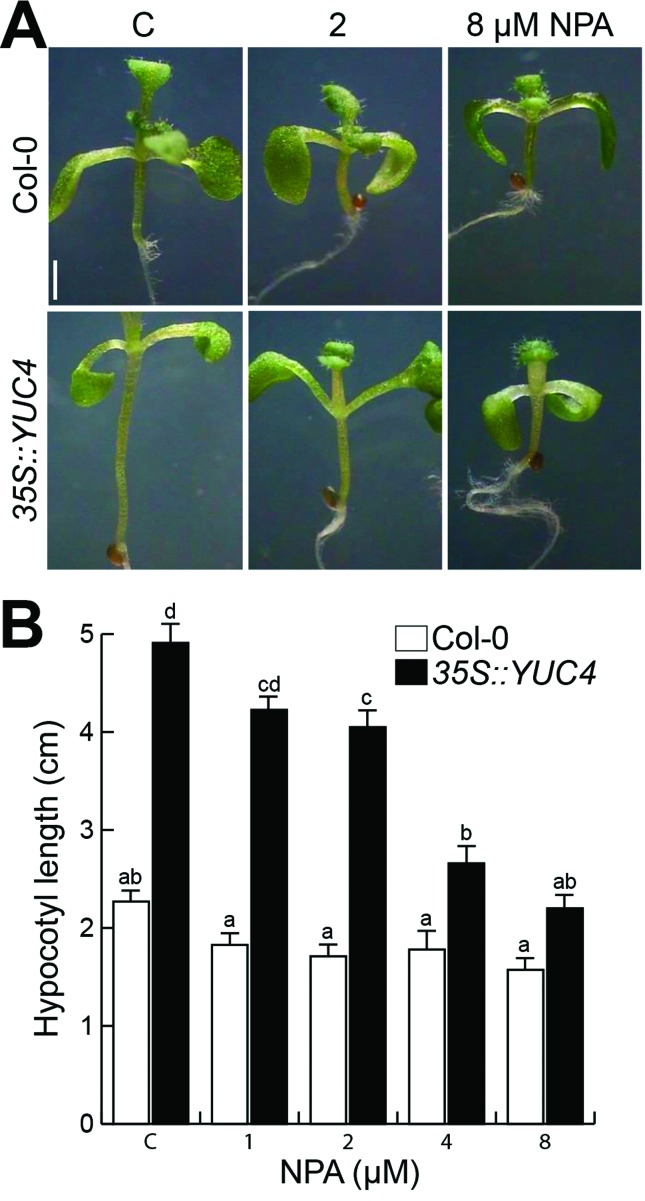
NPA decreases hypocotyl length in *35S::YUC4* seedlings. (A) WT (Col-0) and *35S::YUC4* seedlings were germinated and grown in MS 0.2X media supplemented with different NPA concentrations, representative images of control, 2 and 8 μM of NPA are shown. Bar = 1 mm. (B) Mean hypocotyl length. Error bars represent standard error from 30 seedlings analyzed. Different letters indicate means that are statistically different (*P < 0.05*). The experiment was repeated three times with similar results.

To correlate auxin overproduction and hypocotyl elongation with auxin redistribution as a possible consequence of *YUC4* overexpression, a pharmacological strategy was employed. The response to the auxin transport inhibitor 1-naphthylphthalamic acid (NPA) was compared between WT (Col-0) and *35S::YUC4* seedlings grown side by side in Petri plates containing agar solidified MS 0.2x medium supplemented with DMSO (solvent control) or 1, 2, 4 and 8 μM NPA. After 10 d, the hypocotyl length in WT seedlings remained practically equal in control and NPA treatments. However, in *35S::YUC4* seedlings a dose-dependent shortening of hypocotyls occurred, and at 8 μM NPA *35S::YUC4* hypocotyls were similar to the WT (Figure 4A, 4B). These results suggest that the higher hypocotyl elongation observed in *35S::YUC4* correlates with more auxin being transported to growth zones.

**Figure 5:**
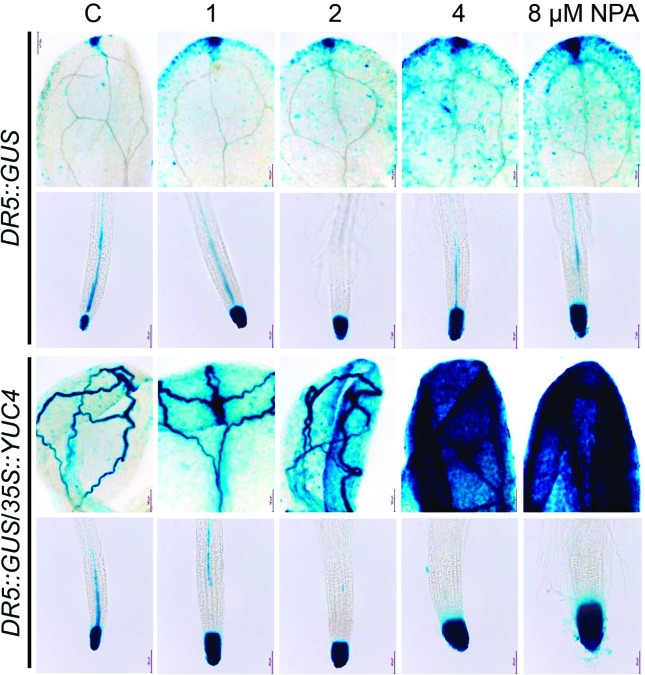
Auxin responsive gene expression is exacerbated in shoots and roots of *35S::YUC4* seedlings upon NPA treatment. *DR5::GUS* expression in WT and *DR5::GUS*/*35S::YUC4* seedlings germinated and grown for 10 d on MS 0.2x medium supplemented with indicated NPA concentrations. Images show representative seedlings for each treatment (n = 15). The seedlings were processed for histochemical detection of GUS expression, cleared, and photographed. Note the dose-dependent exacerbated expression of the marker in *35S::YUC4* seedlings treated with NPA. The experiment was repeated three times with similar results.

To understand how NPA could be affecting overall auxin response and/or distribution, we next compared the expression of *DR5::GUS* reporter construct in leaves and in root tips of WT and *35S::YUC4* seedlings. NPA led to an increased auxin-responsiveness in leaves and root tip of WT seedlings, which was exacerbated in *35S::YUC4* (Figure 5, Figure S3, Figure S4). Another auxin responsive promoter construct, *BA3::GUS* normally expressed in petioles, hypocotyl and slightly in vascular tissues of WT seedlings was also up-regulated in *35S::YUC4* background in a dose-dependent manner (Figure S5). Taken together, these data reinforce the idea that overall auxin accumulation increases as a consequence of *YUC4* overexpression.

### *35S::YUC4* expression up-regulates the ABI4 transcription factor

**Figure 6:**
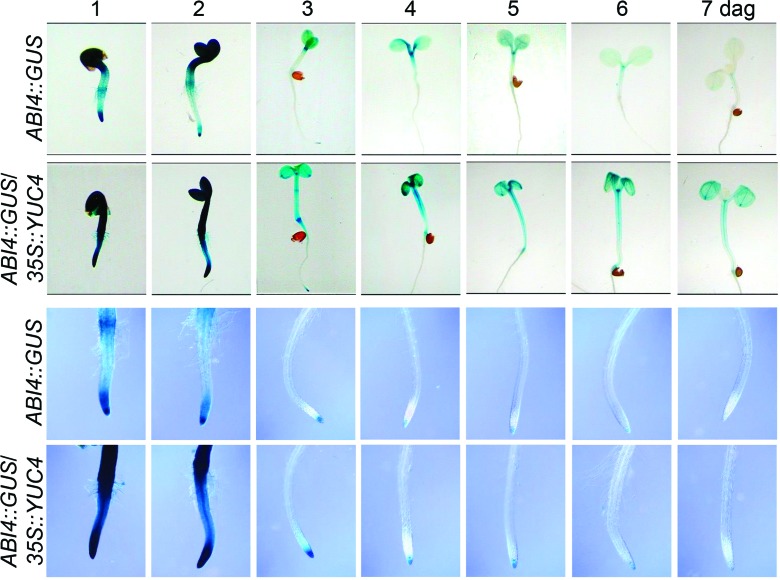
*ABI4::GUS* expression in WT and *35S::YUC4* seedlings. *ABI4::GUS* and *ABI4::GUS*/*35S::YUC4* seedlings were grown for 7 days and histochemical detection of GUS activity performed daily. Photographs show representative hypocotyls and roots of at least 15 stained seedlings. The experiment was repeated three times with similar results.

ABA signaling mediates adaptation to several stressing conditions and also accounts for growth and root architecture modulation (Tiwari *et al.*, 2017). To assess the possible interaction of auxin overproduction in *35S::YUC4* seedlings and ABA signaling, the expression of *ABI4::GUS,* an ABA-related reporter gene that reflects the endogenous *ABI4* transcript level (Bossi *et al.*, 2009; Soderman *et al.*, 2000) was evaluated. GUS expression was monitored 1 to 7 d after germination on *ABI4::GUS* and *35S::YUC4/ABI4::GUS* seedlings grown under standard conditions. GUS expression was evident in WT seedlings since the first day, reaching a maximum by day 2 then gradually decreasing in the subsequent days until practically disappearing on day. Interestingly, in *35S::YUC4* seedlings GUS expression was increased during the kinetic experiment and remained detectable even at day 7 on cotyledons and hypocotyl. In root tips, *ABI4::GUS* expression was also stronger in *35S::YUC4* seedlings than in the WT. However, in both cases it decreased and even disappeared at comparable times (4 and 7 days, respectively; Figure 6). These observations suggest that overexpression of *YUC4* affect ABA signaling through ABI4 at early stages of plant development.

### ABA antagonizes auxin response in WT and *35S::YUC4* seedlings

**Figure 7:**
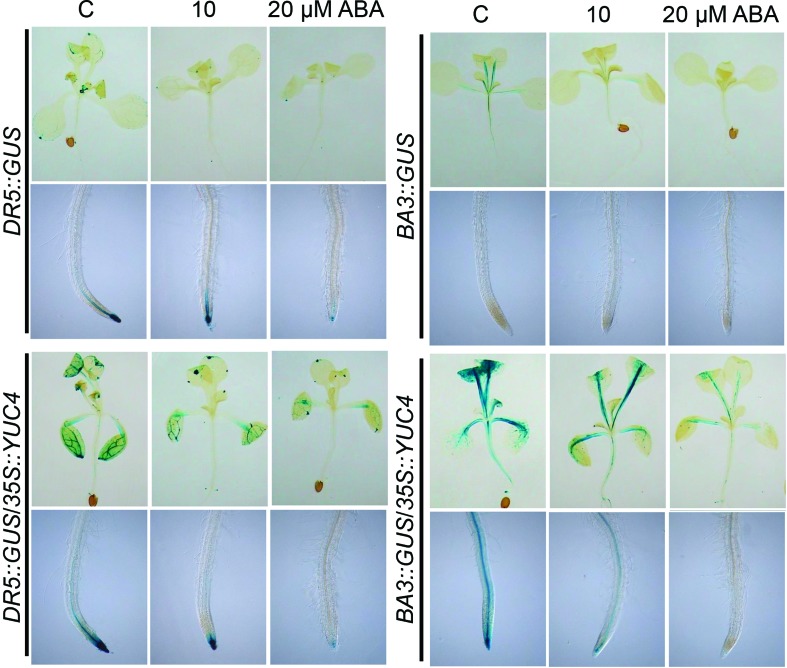
ABA impairs auxin-inducible gene expression in shoots and roots. *DR5::GUS* and *BA3::GUS* gene expression in WT and *35S::YUC4* seedlings germinated and grown for 5 d in agar solidified 0.2x MS medium, then transferred for 5 additional days to fresh medium supplemented with the solvent, 10 or 20 μM ABA. Seedlings were stained for GUS activity and cleared for microscopical analysis. Photographs show representative shoots and roots from at least 15 stained plants. The experiment was repeated three times with similar results.

ABA antagonizes auxin signaling during the formation of lateral roots (De Smet *et al.*, 2003). To test if ABA could affect auxin-inducible gene expression in the shoot and roots systems, the expression of *DR5::GUS* and *BA3::GUS* was assessed in transfer experiments of WT and *35S::YUC4* seedlings grown in medium supplemented with DMSO (control), 10 or 20 μM ABA. An ABA-dependent inhibition of *DR5::GUS* and *BA3::GUS* was clearly observed in the WT and *35S::YUC4* (Figure 7), suggesting that ABA antagonizes auxin responsive gene expression in shoots and in roots.

### *35S::YUC4* seedlings are hypersensitive to ABA

**Figure 8:**
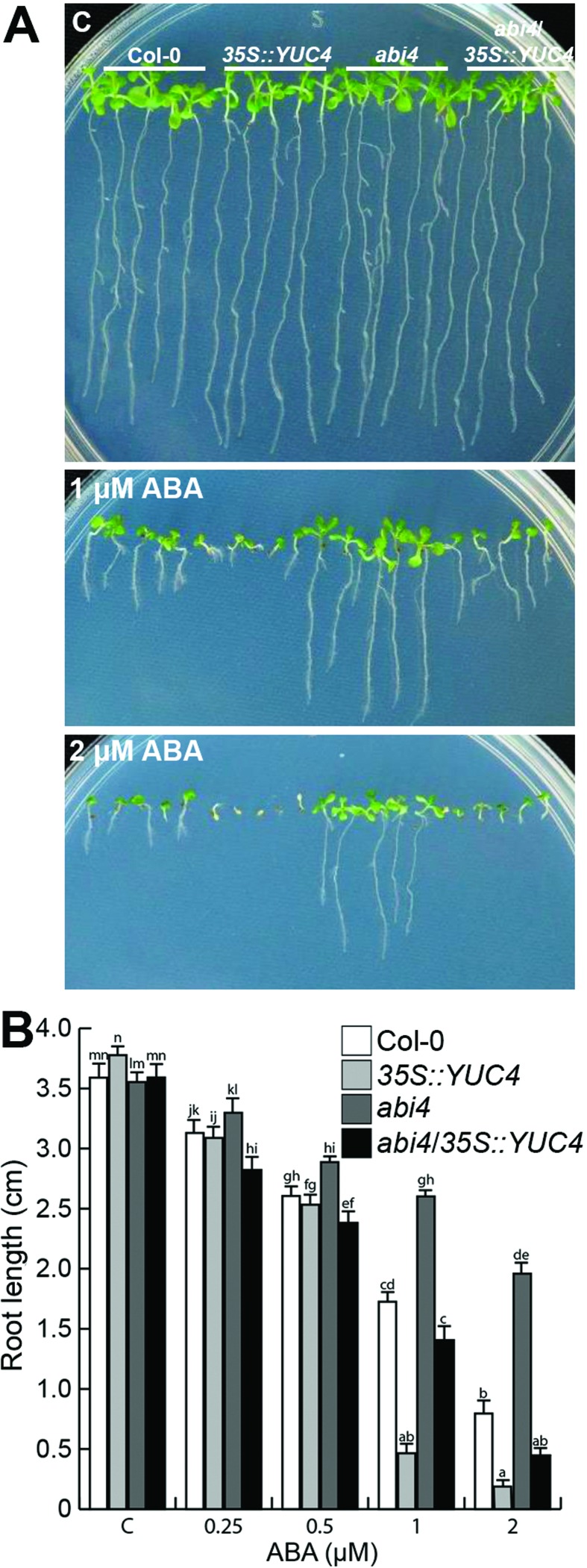
*ABI4* loss of function reduces *35S::YUC4* root hypersensitivity to ABA. (A) Representative images of plates with *Arabidopsis* lines Col-0, *35S::YUC4*, *abi4* and *abi4*/*35S::YUC4* sown on MS media supplemented with the solvent or indicated ABA concentrations. (B) Root length of 6 dag WT, *35S::YUC4*, *abi4* and *abi4*/*35S::YUC4* at 0, 0.25. 0.5, 1 and 2 μM ABA. Error bars represent standard errors from 15 seedlings analyzed and letters indicate means that are statistically different (*P* < 0.05). The experiment was repeated three times with similar results.

ABA inhibits both germination and primary root growth (Gimeno-Giles *et al.*, 2009; Luo *et al.*, 2014), making these responses useful to characterize its potential interaction with auxin via *YUC4*. So, we outcrossed *35S::YUC4* with the *abi4* mutant to further analyze a possible genetic relationship between auxin and ABA signaling mediated by *YUC4*. WT, *35S::YUC4*, *abi4* and *abi4*/*35S::YUC4* seedlings were grown side by side over agar-solidified 0.2x MS medium, supplemented with DMSO or increasing ABA concentrations. Six days after germination plants were analyzed and found that when germinated and grown on control medium, all genotypes behaved similarly (Figure 8A). However, *35S::YUC4* seedlings are hypersensitive to 1 and 2 μM ABA that inhibited primary root growth in the WT, (Figure 8A). As expected, *abi4* mutants were less sensitive to ABA and developed longer primary roots than the WT, whereas *abi4/35S::YUC4* displayed a root length comparable to the WT at higher ABA concentrations.

**Figure 9:**
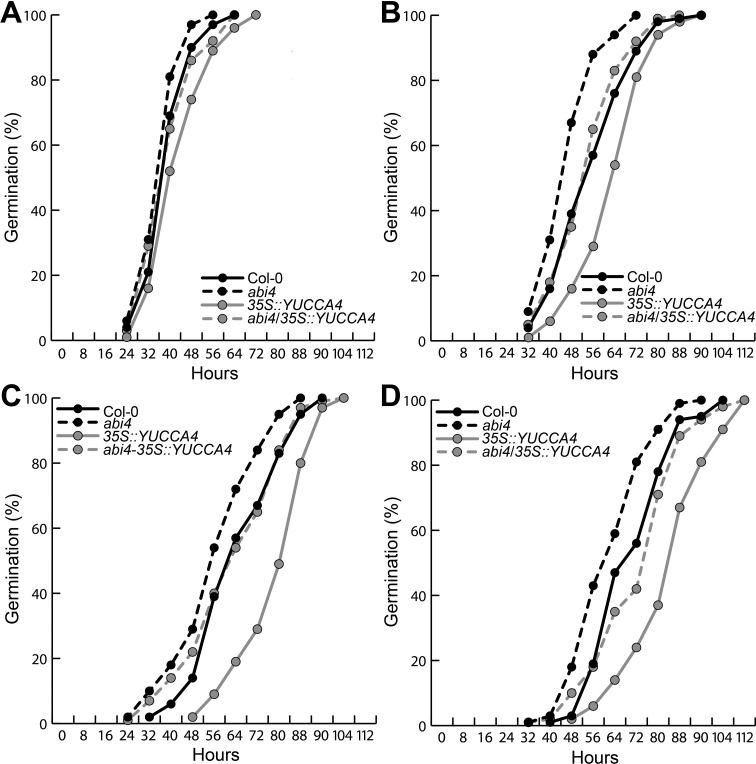
Effects of ABA on germination. *Arabidopsis* seeds of Col-0, *abi4*, *35S::YUC4* and *abi4*/*35S::YUC4* were sown on MS plates containing different ABA concentrations: (A) Mock, (B) 0.5 μM, (C) 1 μM and (D) 2 μM ABA. Radicle protrusion of 100 seedlings of each line was registered every 8 hours. Note the contrasting effects of the ABA over *35S::YUCCA4* and *abi4* seedlings. The experiment was repeated three times with similar results.

To examine the impact that ABA could have on the WT and *35S::YUC4* seedlings on germination, 100 seeds of WT, *35S::YUC4*, *abi4* and *abi4*/*35S::YUC4* were sown on MS plates containing DMSO, or 0.5, 1 and 2 μM of ABA. Plates were placed in darkness and radicle protrusion was evaluated every eight hours until all seeds germinated. In control medium, WT, *abi4* and *abi4*/*35S::YUC4* germinated in around 56 h meanwhile *35S::YUC4* showed a slight delay in germination (Figure 9A). When seeds were sown in medium supplemented with 0.5, 1 and 2 μM ABA, a delayed germination in all four lines already occurred, but interestingly *abi4* and *35S::YUC4* had the opposite performance, germinating earlier or later, respectively, when compared to the WT and *abi4/35S::YUC4* (Figure 9B-D). These results demonstrate the critical role of ABI4 in mediating an auxin-ABA crosstalk for primary root growth and germination.

## Discussion

In this work, five *35S::YUC4 Arabidopsis* lines were characterized, which showed elevated transcript of *YUC4* and up to 25% greater IAA levels than the WT, similar to previous reports in which other members of the *YUC* family were overexpressed (Zhao *et al.*, 2001; Hentrich *et al.*, 2013a). The phenotype observed in *35S::YUC4* included changes in shoots and roots, and were typified by an enhancement of growth. The alterations in root architecture included the formation of longer primary roots with more lateral roots, and to the best of our knowledge, have been not previously reported. Thus, via increasing the endogenous auxin pool more exploratory root systems can be developed.

The auxin-inducible *DR5::GUS* gene construct is expressed in the quiescent center, the adjacent columella cells and root cap (Sabatini *et al.*, 1999). Such expression pattern was found in WT, but in *35S::YUC4* seedlings there was an increased *DR5* induction. In addition, the *HS::AXR3NT-GUS* construct was more rapidly degraded in *35S::YUC4* seedlings. Altogether, these results demonstrate the relationship of the *35S::YUC4* phenotype, degradation of the AUX/IAA AXR3 repressor and the underlying auxin-response in roots and in shoots.

Auxin regulates PIN levels and re-localization (Vieten *et al.*, 2005; Omelyanchuk *et al.*, 2016). In our research, a decreased *PIN1::PIN1-GFP* expression in *35S:YUC4* lines, indicates that auxin overproduction down-regulates PIN1; in contrast, PIN2, PIN3 and PIN7 were up-regulated in *35S::YUC4* seedlings in a tissue-specific context, in concordance with the induction already reported for these transporters by auxin treatment (Vieten *et al.*, 2005; Lewis *et al.*, 2011). A dual role for auxin in the regulation of both PIN transcription and degradation has been proposed, since application of high auxin concentrations decreases *PIN7::GFP* and *PIN2::GFP* signal intensity, whereas at low concentrations, the PIN2 and PIN7 protein amounts are increased (Vieten *et al.*, 2005). Our results show that the *35S:YUC4* significantly increases the endogenous auxin pool, which in roots is high enough to differentially regulate PIN proteins.

Auxin is mainly synthesized in the shoot apex and then transported to the stem and root systems where it regulates growth and tropisms (Spalding, 2013). Although plants that overproduce auxin have long hypocotyls, this effect cannot be mimicked by exogenous application of IAA or synthetic analogs to WT plants. The possibility that an increased auxin transport could be responsible for greater hypocotyl elongation in *35S::YUC4* seedlings is supported from data obtained from the use of NPA, an auxin transport inhibitor, which diminished hypocotyl length in the *YUC4* overexpressors in a dose-dependent manner until the plants attained similar hypocotyl lengths to the untreated WT seedlings. These data suggests that the increased auxin production in the *35S:YUC4* lines inherently changes auxin redistribution, causing elongation of hypocotyls. Consistently, in a recent report NPA antagonized the shade-induced hypocotyl elongation in *Arabidopsis*, presumably because free IAA is prevented from being transported to the growth zones (Zhao, 2018), but also causes IAA accumulation in shoot and root apical meristems (Casimiro *et al.*, 2001; Himanen *et al.*, 2002; Nishimura *et al.*, 2012).

To clarify how auxins are distributed before and after the application of NPA in WT and *35S::YUC4* seedlings, the *DR5::GUS, DR5::GFP* and *BA3::GUS* construct were used. Auxin-driven expression of these constructs was more evident in leaf margins as well as root meristems as a response to NPA treatments, as such *DR5::GUS* histochemical detection was most remarkable in the *35S::YUC4* seedlings, suggesting that auxin accumulates in vascular bundles until filling the whole leaf al high NPA concentrations. Moreover, in the root meristem a characteristic widening of the root was caused by NPA consistent with previous reports (Sabatini *et al.*, 1999; Casimiro *et al.*, 2001), this being more noticeable for *35S::YUC4* seedlings. *BA3::GUS* expression also increased in the *35S::YUC4* lines principally in vascular tissues, and under NPA treatment auxin response exacerbated in leaf veins and root meristem.

The *ABI4* gene encodes an AP2/ERF transcription factor that is expressed in discrete developmental windows, mainly during seed maturation and in young seedlings after germination, during the establishment of autotrophic growth (Finkelstein *et al.*, 1998; Soderman *et al.*, 2000; Arroyo *et al.*, 2003; Shkolnik-Inbar and Bar-Zvi, 2011). Noteworthy, we found an increased expression of *ABI4::GUS* in *35S::YUC4* seedlings. Although it was described that *ABI4* expression is repressed by auxin in roots (Bossi *et al.*, 2009; Shkolnik-Inbar and Bar-Zvi, 2010), the difference with our work is probably due to the different experimental conditions employed; while others exposed plants to high exogenous auxin concentrations during few hours, we tested *ABI4* expression in *35S::YUC4* lines, with moderate and sustained increase in endogenous concentrations of auxin.

Increasing evidence shows that ABA possesses dual functions acting as a growth inhibitor at high concentrations and as a growth promoter at low concentrations. ABA treatment appears to reduce auxin biosynthesis or reduce auxin signaling via decreasing *IAA2,* and concomitantly, *DR5* expression is reduced (Wang *et al.*, 2011; He *et al.*, 2012). We observed that ABA dramatically decreases *DR5::GUS* and *BA3::GUS* expression in shoots and roots of *35S::YUC4* seedlings. This result reinforces the notion of an antagonist role of ABA decreasing auxin biosynthesis, signaling or both these processes.

ABA regulates root elongation through the activities of auxin and ethylene in Arabidopsis (Thole *et al.*, 2014; Rowe *et al.*, 2016). An ABA element involved in root architecture regulation is ABI4; *abi4* mutants develop increased numbers of lateral roots, and *ABI4*-overexpressing plants have a reduced number of lateral roots (Shkolnik-Inbar and Bar-Zvi, 2010). In our experiments, increasing ABA concentrations, delay primary root growth in a dose-dependent manner in WT plants. Moreover a strong hypersensitivity to ABA on seedling growth was observed in *35S::YUC4*, indicating that increased content of endogenous auxin acts in a synergic manner with ABA to repress root growth. On the other hand, *abi4* mutants showed resistance to ABA inhibitory effect, while *abi4*/*35S::YUC4* showed similar root elongation to the WT, demonstrating that *35S::YUC4* hypersensitivity to ABA during early root growth involves the ABI4 transcription factor.

The increased expression of *ABI4::GUS* in *35S::YUC4* seedlings could be an important factor for delayed germination in the auxin overproducing line; to test this, we performed germination assays under increasing ABA concentrations, observing that *35S::YUC4* germinated at a later time in agreement with a previous report, where *Arabidopsis* plants overexpressing *YUC* genes from wheat also underwent delayed germination (Li *et al.*, 2014). Besides, when ABA concentrations increased, delayed germination was more noticeable in *35S::YUC4*; in contrast *abi4* showed resistance to ABA on germination. Previously, *abi4* was shown to be insensitive to auxin and resistant to its combination with ABA during germination (Chen *et al.*, 2014b). Accordingly, in our work, *abi4*/*35S::YUC4* showed similar germination times to the WT, indicating that ABI4 is a required factor for ABA hypersensitivity of *35S::YUC4* during germination, being a convergence element in ABA and auxin mediated control of germination.

The mechanism of interaction between auxins and the ABA is still not fully understood. Previous studies indicate that ABA inhibits seedling growth through enhancing auxin signaling, and the role of auxin signaling elements in ABA responses had been described (Wilson *et al.*, 1990; Fukaki *et al.*, 2002; Tiryaki and Staswick, 2002; Belin *et al.*, 2009; Wang *et al.*, 2011; Rinaldi *et al.*, 2012; Thole *et al.*, 2014). On the other hand, high levels of auxinic compounds enhance the ABA inhibition of germination; besides, ABA elements including ABI3, ABI4 and ABI5 are important regulators of auxin-mediated inhibition of seed germination (Tognetti *et al.*, 2010; Liu *et al.*, 2013; Chen *et al.*, 2014b,c).

Here, we generated a new *35::YUCCA* line to provide more information about physiology of auxin producer plants and we use it as a tool to address the auxin-ABA interaction. Our data strengthen the notion that elevated endogenous auxin levels influence the regulation of seed dormancy, germination and post-embryonic growth in *Arabidopsis,* and functional evidence is provided that *ABI4* is involved in an ABA-auxin interaction important for germination and root growth. The generation and management of knowledge about phytohormone biosynthesis, homeostasis and interactions should assist in developing new tools towards a much needed improvement of certain agronomic traits.

## Supplementary material

The following online material is available for this article:

Figure S1 . IAA levels in roots and shoots of different lines and their crosses with 35S::YUC4.

Figure S2 . Root architecture of 35S::YUC4.

Figure S3 . Quantification of relative GUS expression of DR5::GUS in WT and 35S::YUC4 backgrounds.

Figure S4 . Auxin responsive gene expression is exacerbated in shoots and roots of 35S::YUC4 seedlings upon NPA treatment.

Figure S5 . Auxin-inducible BA3::GUS expression in response to NPA treatment.
