# Supplementary material for: YUCCA4 overexpression modulates auxin biosynthesis and transport and influences plant growth and development via crosstalk with abscisic acid in Arabidopsis thaliana
Source: Genet Mol Biol. 2020 Feb 17;43(1):e20190221. doi: 10.1590/1678-4685-GMB-2019-0221 (PMC7197984; doi:10.1590/1678-4685-GMB-2019-0221)
Supplement: Supplementary file 1 [file 1415-4757-GMB-43-1-e20190221-suppl1.pdf]

Supplementary Material to "*YUCCA4* overexpression modulates auxin biosynthesis and transport and influences plant growth and development via crosstalk with abscisic acid in *Arabidopsis thaliana*"

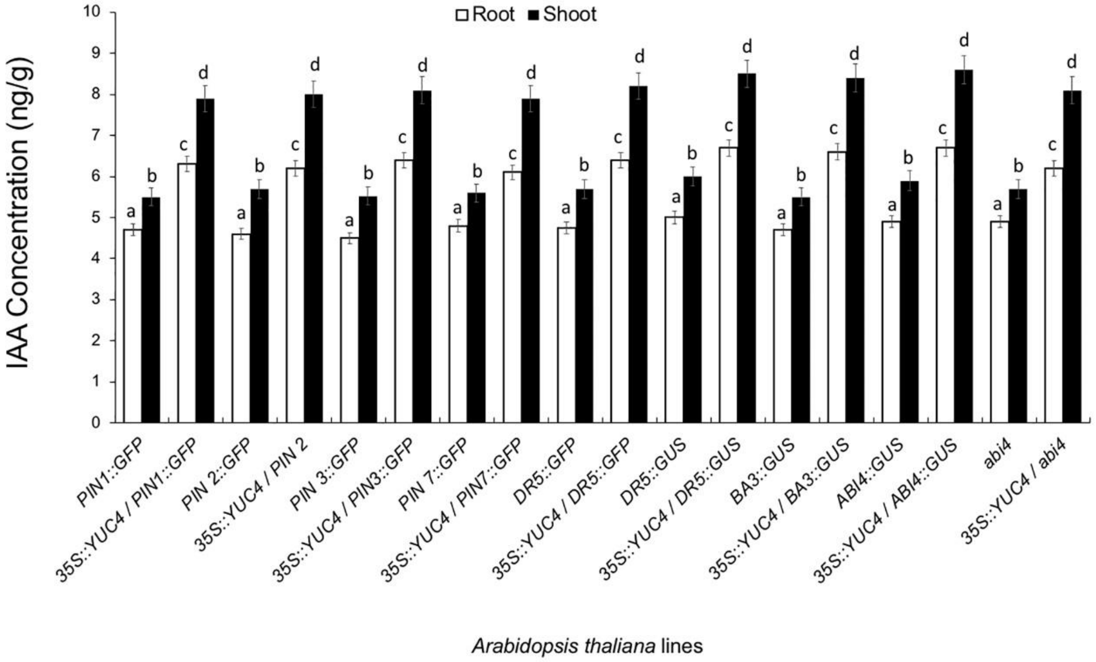

Figure S1 - IAA levels in roots and shoots of different lines and their crosses with *35S::YUC4*. Seedlings of reporter lines and mutants used in this work and their respective crosses with *35S::YUC4* were grown on 0.2x MS medium for 10 d, then IAA was determined by GC-MS as described in materials and methods section. Bars show standard errors and different letters indicate statistical differences at $P = 0.05$.
