# Supplementary material for: YUCCA4 overexpression modulates auxin biosynthesis and transport and influences plant growth and development via crosstalk with abscisic acid in Arabidopsis thaliana
Source: Genet Mol Biol. 2020 Feb 17;43(1):e20190221. doi: 10.1590/1678-4685-GMB-2019-0221 (PMC7197984; doi:10.1590/1678-4685-GMB-2019-0221)
Supplement: Supplementary file 2 [file 1415-4757-GMB-43-1-e20190221-suppl2.pdf]

# Supplementary Material to "*YUCCA4* overexpression modulates auxin biosynthesis and transport and influences plant growth and development via crosstalk with abscisic acid in *Arabidopsis thaliana*"

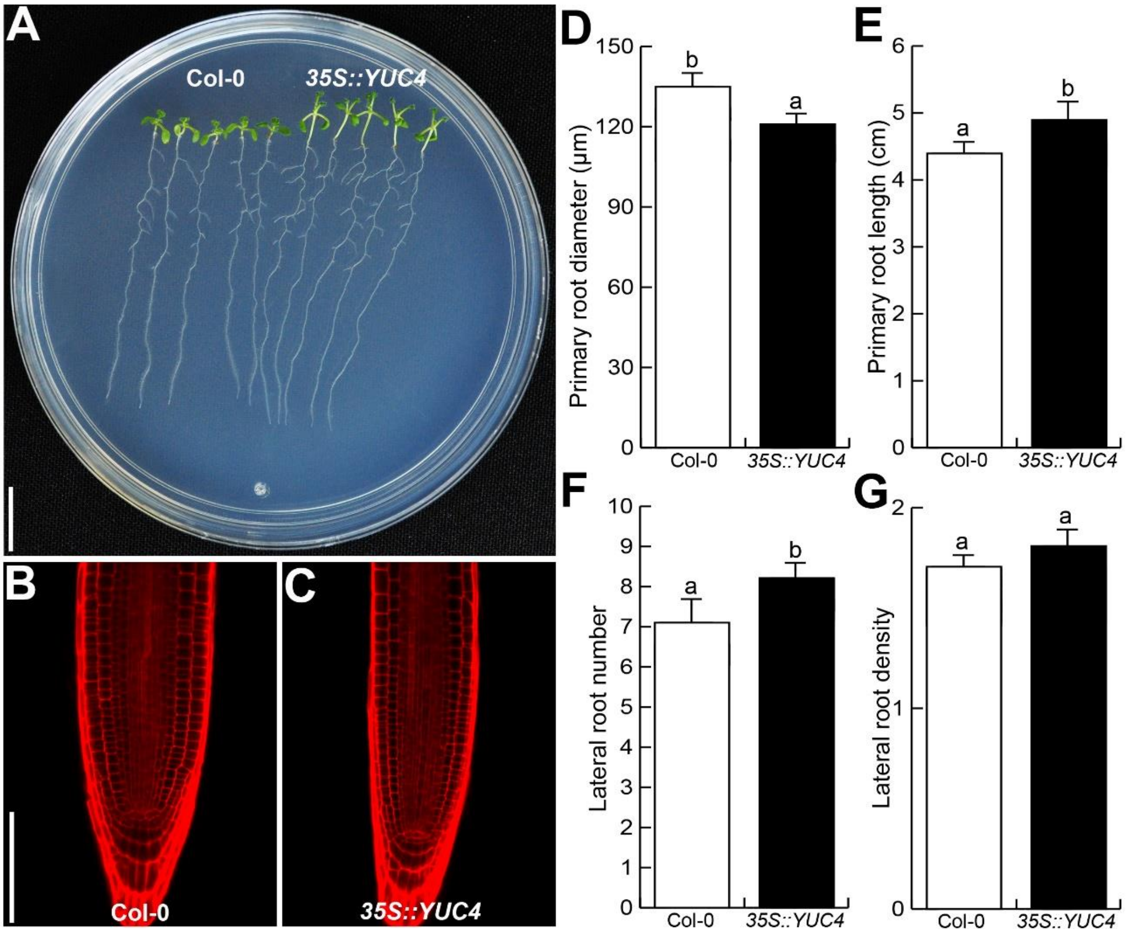


**Figure S2 -** Root architecture of *35S::YUC4*. (A) Representative image of WT (Col-0) and *35S::YUC4* seedlings grown side by side for 10 d; bar = 1 cm. (B) and (C) confocal images of root meristems of WT and *35S::YUC4* stained with propidium iodide; bar = 100 µm. D-G Quantification of primary root diameter (D), primary root length (E), lateral root number (F), and lateral root density (G) of WT and *35S::YUC4* seedlings. Bars indicate standard error (n = 10), and different letters indicate means that are statistically different (*P = 0.05*).
