# Supplementary material for: YUCCA4 overexpression modulates auxin biosynthesis and transport and influences plant growth and development via crosstalk with abscisic acid in Arabidopsis thaliana
Source: Genet Mol Biol. 2020 Feb 17;43(1):e20190221. doi: 10.1590/1678-4685-GMB-2019-0221 (PMC7197984; doi:10.1590/1678-4685-GMB-2019-0221)
Supplement: Supplementary file 3 [file 1415-4757-GMB-43-1-e20190221-suppl3.pdf]

Supplementary Material to "*YUCCA4* overexpression modulates auxin biosynthesis and transport and influences plant growth and development via crosstalk with abscisic acid in *Arabidopsis thaliana*"

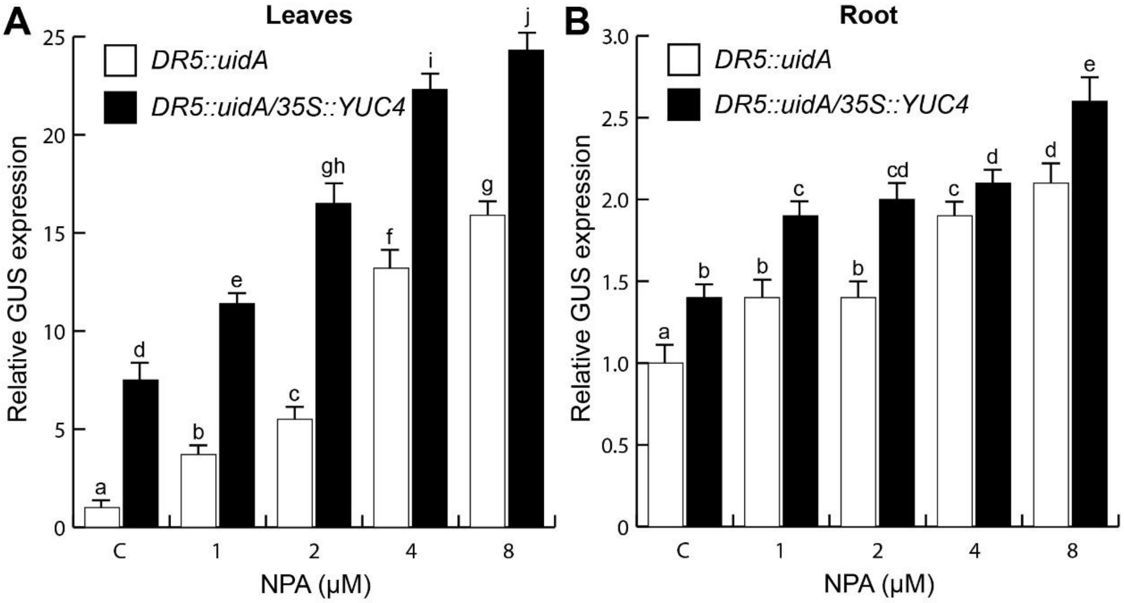

Figure S3 - Quantification of relative GUS expression of *DR5::GUS* in WT and *35S::YUC4* backgrounds. Plants were grown on MS 0.2X supplemented with increased NPA concentrations, at 10 dag were stained and photographed. Relative GUS expression was determined from leaves (A) and root meristem (B) using imageJ software (n = 10). Bars in graphics indicate standard error and different letters indicate statistical differences at $P = 0.05$.
