# Supplementary material for: YUCCA4 overexpression modulates auxin biosynthesis and transport and influences plant growth and development via crosstalk with abscisic acid in Arabidopsis thaliana
Source: Genet Mol Biol. 2020 Feb 17;43(1):e20190221. doi: 10.1590/1678-4685-GMB-2019-0221 (PMC7197984; doi:10.1590/1678-4685-GMB-2019-0221)
Supplement: Supplementary file 4 [file 1415-4757-GMB-43-1-e20190221-suppl4.pdf]

Supplementary Material to "*YUCCA4* overexpression modulates auxin biosynthesis and transport and influences plant growth and development via crosstalk with abscisic acid in *Arabidopsis thaliana*"

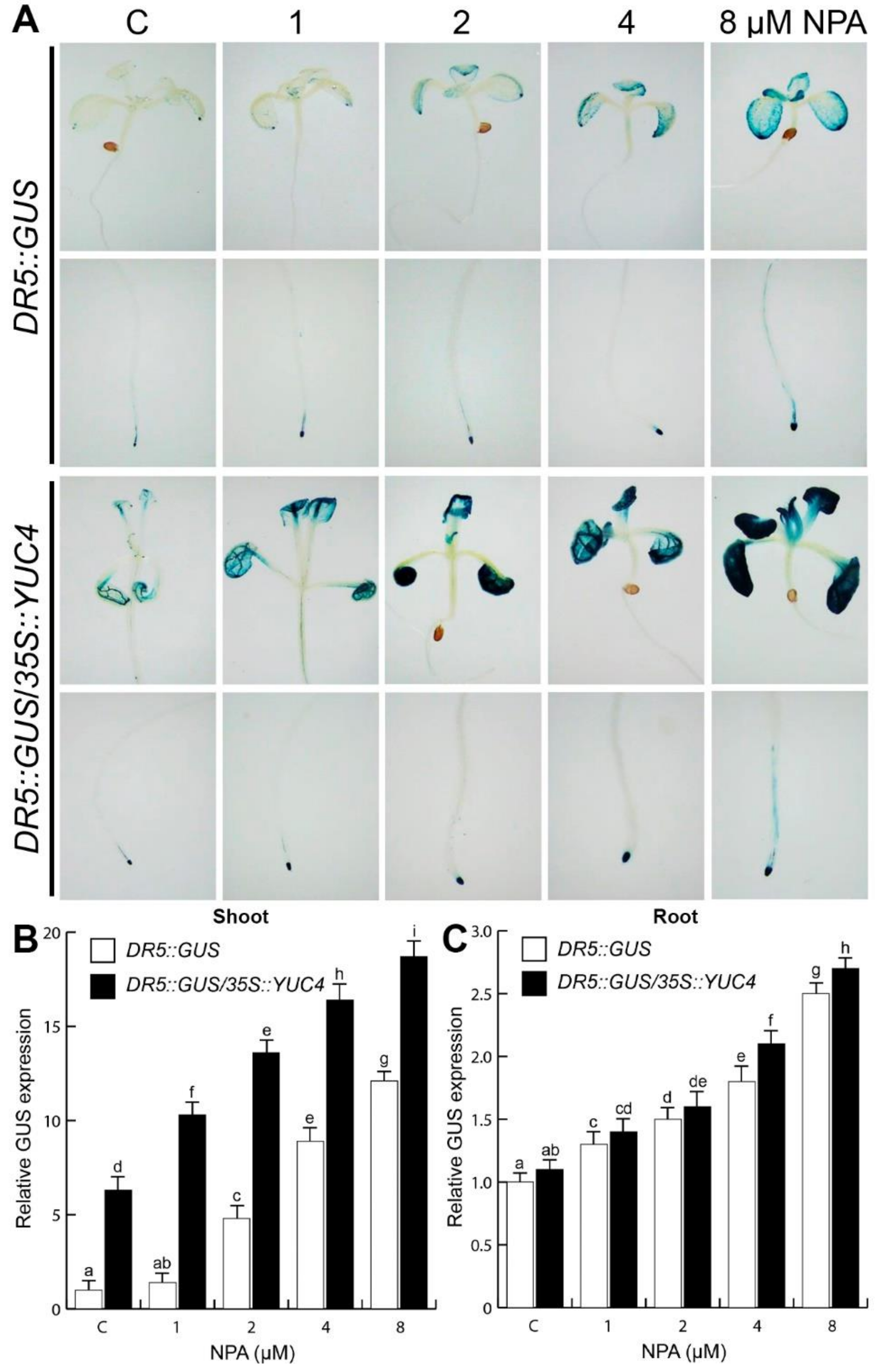

Figure S4 - Auxin responsive gene expression is exacerbated in shoots and roots of *35S::YUC4* seedlings upon NPA treatment. *DR5::GUS* expression in WT and *DR5::GUS/35S::YUC4* seedlings germinated and grown for 10 d on MS 0.2x medium supplemented with indicated NPA concentrations. Images in (A) show representative seedlings for each treatment (n = 15). Note the dose-dependent exacerbated expression of the marker in *YUC4* overexpressing seedlings treated with NPA. The relative expression of the marker gene in shoots (B) and roots (C) of WT and *35S::YUC4* backgrounds was determined using the imageJ software analyzing images from each treatment (n = 10). Bars indicate standard error and different letters indicate statistical differences at *P = 0.*
