# Supplementary material for: YUCCA4 overexpression modulates auxin biosynthesis and transport and influences plant growth and development via crosstalk with abscisic acid in Arabidopsis thaliana
Source: Genet Mol Biol. 2020 Feb 17;43(1):e20190221. doi: 10.1590/1678-4685-GMB-2019-0221 (PMC7197984; doi:10.1590/1678-4685-GMB-2019-0221)
Supplement: Supplementary file 5 [file 1415-4757-GMB-43-1-e20190221-suppl5.pdf]

Supplementary Material to "*YUCCA4* overexpression modulates auxin biosynthesis and transport and influences plant growth and development via crosstalk with abscisic acid in *Arabidopsis thaliana*"

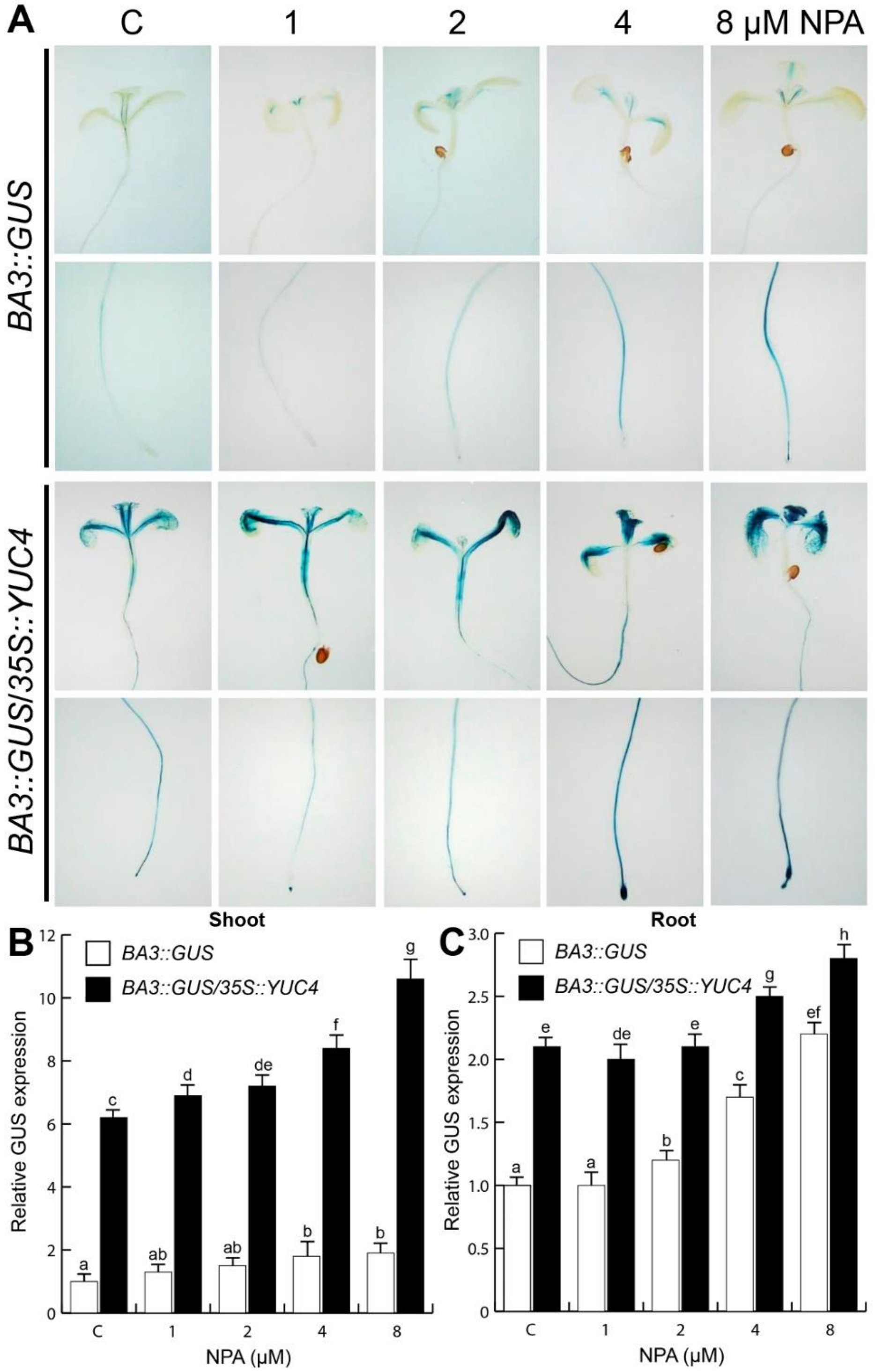

Figure S5 - Auxin-inducible *BA3::GUS* expression in response to NPA treatment. *BA3::GUS* expression in WT and *35S::YUC4* seedlings that were grown in medium supplemented with increasing NPA concentrations. 10 d seedlings were processed for histochemical detection of GUS activity, cleared and photographed. Representative seedlings for each treatment are showed in (A) (n = 15). The relative expression of the marker gene in shoots (C) and roots (D) was determined for both backgrounds using the imageJ software (n = 10). Bars indicate standard error and different letters indicate statistical differences at $P = 0.05$.
